# Etude des facteurs liés à l'observance au traitement antirétroviral chez les patients suivis à l'Unité de Prise En Charge du VIH/SIDA de l'Hôpital de District de Dschang, Cameroun

**Published:** 2012-06-29

**Authors:** François-Xavier Mbopi-Kéou, Lucienne Dempouo Djomassi, Francisca Monebenimp

**Affiliations:** 1 Laboratoire National de Santé Hygiène Mobile, Ministère de la Santé Publique, Yaoundé, Cameroun et Faculté de Médecine et des Sciences Biomédicales, Université de Yaoundé I, Yaoundé, Cameroun; 2 Direction de la Lutte contre la Maladie, Ministère de la Santé Publique, Yaoundé, Cameroun; 3 Centre Hospitalier Universitaire de Yaoundé et Faculté de Médecine et des Sciences Biomédicales, Université de Yaoundé I, Yaoundé, Cameroun

**Keywords:** Observance, VIH/SIDA, traitement antiretroviral, Cameroun

## Abstract

**Introduction:**

Etudier les facteurs liés à l'observance au traitement antirétroviral chez les patients adultes suivis à l'Unité de Prise en Charge du VIH/SIDA (UPEC) de l'hôpital de District de Dschang.

**Méthodes:**

Dans une étude descriptive transversale conduite à l'hôpital de District de Dschang, l'observance a été évaluée sur la base des déclarations des patients et sur la régularité du renouvellement de leurs ordonnances (observance calculée).

**Résultats:**

Parmi les 389 patients répondant à nos critères d'inclusion, 356 ont été interrogés. La durée moyenne du suivi était de 27 mois. La moyenne d’âge était égale à 41 ans et le sexe ratio 2,46 en faveur du sexe féminin. Le statut sérologique était découvert pour 60,56% des patients à l'occasion d'un épisode maladif. Le niveau d'observance déclarée était significativement plus élevé que le niveau global de l'observance calculée (80,2% vs 51,5%, p<10^−5^). Les deux principales barrières à l'observance étaient l'oubli et le travail. Les patients référés dans cette UPEC étaient moins bien observants (p<10^−4^). L'observance au traitement antirétroviral était d'autant meilleure quand le taux de CD4 en début de traitement était élevé (p= 0,01) et que la durée du traitement était prolongée (p=0,00).

**Conclusion:**

La discordance observée entre les résultats des deux méthodes utilisées pour estimer l'observance, tout en soulévant les contraintes liées à l’évaluation de l'observance thérapeutique, souligne l'importance des méthodes biologiques. Les facteurs individuels se sont avérés être les principales raisons de non-observance. Enfin, un accent devrait être mis sur les consultations d’éducation thérapeutique et le suivi psycho-social des patients sous traitement antirétroviral dans cette UPEC.

## Introduction

Avant la découverte des antirétroviraux, on ne disposait d'aucun médicament actif contre le virus de l'immunodéficience humaine (VIH). L′introduction des combinaisons thérapeutiques hautement actives en 1996 a bouleversé l′évolution naturelle de cette infection en induisant une baisse effective de la morbidité et de l'incidence de nouveaux cas de sida [1]; elle a changé le pronostic des malades infectés en conférant à cette pathologie un caractère chronique [2]. Cependant, très rapidement des difficultés liées à la lourdeur du traitement, aux effets secondaires multiples et donc aux problèmes d'observance sont apparues, compromettant alors le succès du traitement antirétroviral (TAR).

L'observance peut être définie comme « le degré d′adéquation entre le comportement du patient en terme de prise de médicament, et les prescriptions et recommandations médicales » [3]. Elle constitue avec l'absence de traitement antirétroviral antérieur les principaux facteurs du succès du TAR [4–6], élément fondamental de la riposte à l'infection par le VIH [7]. Une observance du traitement de 95% est nécessaire [8, 9] pour atteindre les objectifs thérapeutiques qui sont: prolonger la vie, diminuer la fréquence des affections opportunistes, arrêter ou ralentir rapidement et durablement la réplication virale, restaurer ou améliorer l'immunité de la personne infectée [10–13]. Dans la littérature, la mesure de l'observance est le plus souvent basée sur des questionnaires destinés à évaluer l'observance des patients au cours des derniers jours. Des questionnaires ciblant les prescripteurs ont aussi été utilisés; toutefois, leur fiabilité s'est avérée limitée [14]. L'observance a également été appréciée par le comptage des comprimés restants dans les systèmes o[ugrave] le patient est appelé á s'approvisionner en médicaments auprès d'une même pharmacie. L'utilisation des piluliers électroniques est très souvent utilisée dans des études pilotes, en particulier lorsque le nombre de patients n'est pas important. Enfin, le taux de renouvellement des ordonnances est apparu être un bon indicateur d'observance, sensible à la durée ou la complexité du TAR [15].

Des études menées jusqu'ici ne font pas état d'un profil socio-économique des patients a priori non-observants au Cameroun. Orrell et al dans une étude menée à Cape Town de Janvier 1996 à Mai 2001 ont retrouvé un niveau d'observance (93,5%) comparable à celui observé dans les pays développés dans une cohorte de 289 patients infectés par le VIH et vivant dans une extrême pauvreté [16]. A Dakar, Isabelle Laniece et al ont mesuré l'observance par la méthode de comptage des restes de médicaments à chaque visite, sur une période de 12 mois sur un échantillon de 158 adultes infectés par le VIH et ont retrouvé un niveau d'observance de 91% [17].

Avec une séroprévalence estimée à 5,4% au sein de la population adulte [18], le gouvernement camerounais a fait de la lutte contre le SIDA l'un des axes prioritaires de sa politique de développement. La riposte nationale à ce jour, a consisté en un éventail d'interventions allant de la prévention à la gratuité du TAR (en vigueur depuis le 1er Mai 2007), en passant par la baisse des coûts annexes liés aux examens complémentaires et à la prise en charge de certaines infections opportunistes, la protection et le soutien aux personnes affectées par le VIH/SIDA, la promotion de la recherche et la surveillance épidémiologique. Toutefois, cette stratégie a été très rapidement confrontée aux insuffisances du système de santé liées à l'approvisionnement et à la gestion des stocks. Ainsi, la pénurie de certaines combinaisons thérapeutiques et réactifs de laboratoire n'est pas inhabituelle dans les formations sanitaires camerounaises, contrariant d'ores et déjà la qualité de la prise en charge et le suivi des patients.

L'hôpital de district de Dschang (HDD) est la formation sanitaire de référence dans le District de Santé (DS) de Dschang, Région de l'Ouest Cameroun. Il possède en son sein une Unité de Prise en Charge (UPEC) des personnes vivant avec le VIH/SIDA. Les activités de dépistage, de prévention et de prise en charge sont intégrées aux différents services de soins. Seules les multi thérapies de première ligne (2 INTI+ 1 INNT, 3 INTI) sont disponibles dans la pharmacie, les cas nécessitant les secondes lignes thérapeutiques sont référés au Centre de traitement agrée de Bafoussam (Chef-lieu de région). Tous les malades sont appelés à se présenter chaque mois à la pharmacie pour le renouvellement de leurs ordonnances et au moins une fois tous les trois mois chez l'un des 6 médecins habilités à prescrire les antirétroviraux (ARV) pour leur suivi médical. Le soutien psychosocial est assuré principalement par des membres des associations de personnes vivant avec le VIH (PVVs) implantées dans la localité, les agents de relais communautaires (ARC), l'assistante sociale et dans une certaine mesure par le personnel soignant formé au conseil et sur l'aide à l'observance.

Dans ce travail, nous nous proposons d’étudier les facteurs liés à l'observance du traitement antirétroviral chez la cohorte de patients adultes suivis à l'Hôpital de District de Dschang. Plus spécifiquement, il s'agit pour nous de: décrire les caractéristiques sociodémographiques des patients adultes sous TAR suivis à l'HDD; déterminer leur niveau d'observance du traitement antirétroviral; identifier les déterminants de l'observance et les raisons de non-observance du TAR chez ces patients.

## Méthodes

### Schéma d’étude

Il s'agissait d'une étude descriptive transversale conduite du 4 Mai au 26 Juin 2010 à l'HDD.

### Population d’étude

Elle était constituée des patients âgés de plus de 18 ans, sous TAR et suivis depuis au moins trois mois à l'UPEC de l'HDD. Notre échantillon était constitué des sujets non hospitalisés venus pour le renouvellement mensuel de leurs ordonnances ou pour leurs visites médicales de routine à l'UPEC de l'HDD et auprès desquels l'accord de participation avait été obtenu par un consentement éclairé et écrit.

### Recueil des données

Il était systématiquement proposé à chaque patient venu renouveler son ordonnance, de participer à l'enquête. Le questionnaire utilisé s'intéressait aux caractéristiques socio-démographiques des patients, aux connaissances sur la transmission du virus et la prévention de l'infection par le VIH/SIDA, aux circonstances de diagnostic du statut sérologique et à la classification CDC de 1993 en début de traitement, aux différents recours thérapeutiques et à l'observance thérapeutique au TAR. L'analyse des registres de la pharmacie a permis de recueillir pour la population d’étude, les informations relatives à la date de début de traitement, au statut vis-à-vis de la référence, au stade clinico-biologique en début de traitement et à la fréquence d'approvisionnement en ARV. Celle des dossiers médicaux permettait de confirmer les informations sociodémographiques, les circonstances de découverte du diagnostic sérologique et de relever le taux de CD4 en début de traitement. Les entretiens étaient conduits en Français et/ou en langue maternelle par des enquêteurs préalablement formés. Il s'agissait des Agents de Relais Communautaire (ARC), répondants des services de santé au niveau de la communauté et jouant le rôle d'interface entre les professionnels de la santé et les populations locales.

### Analyse des données

La population d’étude a été décrite, et les caractéristiques des patients observants ont été comparées à ceux des patients non observants par les tests de Khi2 ou de Fisher pour les variables qualitatives et l'analyse par ANOVA ou le test non paramétrique de Mann Whytney/Wilcoxon pour les variables quantitatives. Cette analyse comparative a été complétée par une analyse multivariée par régression logistique, la variable d'intérêt étant l'observance. Etaient considérés comme perdus de vue les patients ne s’étant pas présentés à la pharmacie de l'hôpital depuis au moins 3 mois.

Le niveau général de connaissance a été apprécié par un score, sommant les différents items évoqués dans la rubrique connaissance sur mode de transmission et prévention du VIH (boîte 1); il était coté sur 11, et étalonné sur une échelle à trois grades: faible (0–5); moyen (6–8); bon (9–11)

L'observance thérapeutique a été évaluée à l'aide de 3 items:

**Observance déclarée:** appréciée par la fréquence de saut de prise de médicaments depuis l'initiation thérapeutique. Ont alors été définis non observants: Les sujets ayant sauté plus d'une prise de médicaments au cours des 7 derniers jours pour les protocoles à trois prises journalières (soit moins de 95% des prises absorbées); Les sujets ayant sauté plus d'une prise de médicaments durant les 10 derniers jours pour les protocoles à deux prises quotidiennes (soit moins de 95% des prises absorbées); Les personnes ayant sauté pendant une semaine ou plus leur traitement depuis l'initiation thérapeutique [19]. **Observance calculée:** mesurée par le rapport entre le nombre d'ordonnances dispensées et la quantité théorique d'ordonnances attendues (lequel correspond au nombre de mois de suivi du traitement). Le patient était considéré comme observant lorsque ce coefficient était supérieur à 0,95. **Le jugement global** que le patient se faisait sur le respect des horaires de prise du traitement.

La saisie des données et l'analyse statistique ont été faites à l'aide du logiciel EpiInfoTM version 3.5.1.

## Résultats

### Exhaustivité des données

Parmi les 389 patients éligibles à l'enquête, 356 ont été interrogés, soit une exhaustivité globale de 91,5%. 10 patients étaient perdus de vue, 6 étaient hospitalisés au moment du renouvellement de leurs ordonnances, 12 ont refusé de participer et 5 ne se sont pas présentés à la pharmacie durant la période d’étude. L'analyse des dossiers médicaux des patients n'ayant pas participé à l’étude n'a pas révélé de différence significative avec ceux ayant participé pour ce qui est des caractéristiques sociodémographiques et clinico-biologiques (Tableau 1).

**Tableau 1:** Quelques caractéristiques démographiques et clinico-biologiques des patients suivis depuis plus de trois mois à l'Unité de Prise en Charge du VIH/SIDA de Dschang, Ouest Cameroun

Variables	Éligibles Enquêtés		Éligibles non enquêtés		P value
	N=356	%	N=33	%	
**Age**					0,99
≤30	46	12,9	4	11,2	
31–50	237	66,6	22	68,3	
51–60	62	17,4	6	17,6	
>60	11	03,1	1	2,9	
**Sexe**					0,78
Féminin	253	71,1	22	69,2	
Masculin	103	28,9	10	33,8	
**Religion**					0,55
Chrétienne	243	68,6	21	63,6	
Animiste	111	31,4	12	36,4	
Musulmane	0	0,0			
Manquant	2				
**Statut matrimonial**					0,69
Célibataire	53	15,0	5	16,7	
Divorcé (e)	27	7,7	2	6,7	
En séparation	19	5,4	0	0	
En cohabitation	9	2,5	0	0	
Marié (e)	143	40,5	12	40,1	
Veuf (ve)	102	28,9	11	36,5	
Manquant	3		3		
**Niveau de scolarisation**			NR		–
Non scolarisé	13	3,7			
Primaire	160	46,6		NR	
Secondaire	150	43,8			
Post-secondaire	20	5,9			
Manquant	13				
**Catégorie socioprofessionnelle**					0,94
Agriculture	98	27,5	10	29,4	
Aucune/Retraite	96	27,0	9	26,8	
Commerce	51	14,3	5	15,6	
Enseignement	21	5,9	2	5,6	
Transport	13	3,6	1	3,4	
Coiffure	12	3,4	1	3,9	
Téléphonie mobile	8	2,3	0	0	
Couture	7	2,0	0	0	
Etudiant	4	1,1	0	0	
Maçonnerie	4	1,1	0	0	
Menuiserie	4	1,1	0	0	
Mécanique auto	3	0,8	0	0	
Ingénierie	2	0,6	0	0	
Aide-ménagère	2	0,6	0	0	
Soins infirmiers	1	0,3	0	0	
Autres	30	8,4	5	15,3	
**Stade CDC**					0,81
A	187	54,5	16	52,7	
B	139	40,4	14	43,2	
C	18	5,1	1	4,1	
Manquant	12		2		
**Protocole thérapeutique**					0,67
2 INTI + Efavirenz	76	21,4	6	18,6	
2 INTI + Névirapine	280	78,6	27	81,4	
**CD4 à l'initiation (Cellules/ml)**	**N=342**		**N=28**		0,14
Premier quartile	60		55		
Médiane	108		102		
Troisième quartile	169		171		

* NR : Non renseigné

### Caractéristiques socio-démographiques

Ils sont résumés dans le Tableau 1. L′âge moyen des patients était de 41 ans. La tranche d’âge de 31–50 ans étaient prédominante (66,6%) et 3,1% des sujets avaient plus de 60 ans. La médiane d’âge était plus élevée chez les hommes que chez les femmes (44 ans vs 37 ans, p<10-5). Le sexe ratio était de 2,46 femmes pour 1 homme.

Un peu plus de la moitié des sujets répondants (57%) vivaient seuls (célibataire, en séparation, divorcé, veuf). Environ deux tiers d'entre eux confessent la foi chrétienne (68,6%). Parmi les personnes interrogées, 90,4% avaient un niveau de scolarité primaire ou secondaire.

L'agriculture était la principale activité pratiquée (27,5%); un peu plus d'un quart d'entre eux (27%) n'exerçaient aucune activité professionnelle ou alors étaient retraités. Enfin, 85,80% des sujets répondants résidaient à l'intérieur du district.

### Connaissances sur la transmission et la prévention du VIH

Les sujets de cette cohorte étaient relativement bien informés sur les modes de transmission du virus et les moyens de prévention de la maladie (boîte 1). 78.9% ont cité au moins 4 modes de transmission et 99,7% ont cité au moins un. 76,1% ont cité au moins 3 moyens de prévention et 99,4% au moins un. 5,9% des sujets pensaient que le VIH peut se transmettre par l'un des moyens suivants: voie mystique, vêtement, piqûre de moustique, toux, salive, seau de bain. Le niveau général de connaissance était bon dans 64% des cas (IC: 58,8%; 69,0%) et moyen dans 17,7% des cas (IC: 14,0%; 22,2%).

### Caractéristiques clinico-biologiques

**Figure 1:**
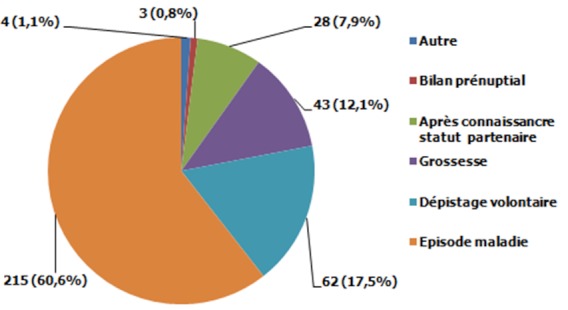
Circonstances de diagnostic de la séropositivité : Cohorte de patients suivis depuis plus de trois mois à l'Unité de Prise en Charge du VIH/SIDA de Dschang, Ouest Cameroun

La découverte de la séropositivité a eu lieu au décours d'un épisode maladif (Graphique 1) pour deux tiers d'entre eux (60,56%). Chez les sujets de sexe féminin, le bilan prénatal avait permis de révéler le statut séropositif pour 17% d'entre elles. La moyenne CD4 à l′initiation était de 104; le mode étant 96. Les stades clinico-immunologiques selon la classification CDC (1993) en début de traitement étaient principalement A3 (54,5%) suivi de B3 (40,4%) (Figure 1). Environ, la moitié des sujets recherchaient le soutien émotionnel et psychologique auprès de leurs amis (50,9%). Pour ceux d'entre eux qui vivaient en union (libre ou légale), le/la conjoint (e) constitue la première source de soutien émotionnel et psychologique (43,3%).

### Le dépistage volontaire et le conseil

Le dépistage volontaire était la deuxième circonstance de diagnostic la plus fréquente (17,46%). Le conseil pré-thérapeutique avait été administré pour 95,8% des sujets, plus souvent par un ARC (51,88%) qu'un médecin (35,07%).

### Le traitement

La mise sous ARV avait été décidée par le comité thérapeutique de l'UPEC de Dschang dans 70,8% des cas, les autres patients de la cohorte (29,2%) étant référés vers cette UPEC à partir d'un autre centre de traitement pour suivi thérapeutique. Le régime thérapeutique le plus fréquemment prescrit était l'association Lamividune-Stavudine-Névirapine (70,8%). Les combinaisons thérapeutiques incluant l'Efavirenz représentaient 21,4% des cas. La durée moyenne du suivi est 29 mois pour les patients transférés et de 25 mois pour les non transférés; soit une moyenne pondérée de 27 mois.

- **La place de la médecine traditionnelle:** Le recours à la médecine traditionnelle avant le début du TAR était rapporté par 29,2% des sujets; 86% d′entre eux avaient déclaré ne plus visiter les tradipraticiens depuis la mise en route du traitement et 9,1% anticipaient les consulter dans le futur, la recherche de la guérison définitive étant la principale motivation. En analyse univariée, les patients ayant eu recours au traitement traditionnel après le début du TAR apparaissaient moins observants (Tableau 2 et Tableau 3) que ceux sous TAR exclusif. Toutefois, après ajustement sur les déterminants significatifs de l'observance observés lors de l'analyse univariée, cet effet de la médecine traditionnelle sur l'observance au TAR s'estompait (Tableau 4)

**Tableau 2:** Observance calculée selon les caractéristiques socio-démographiques et clinico-biologiques, Unité de Prise en Charge du VIH/SIDA de Dschang, Ouest Cameroun

Variables qualitatives	Observants (%)	Non-observants (%)	P-value
**Sexe (N=356)**			
Masculin	70,9	29,1	0,83
Féminin	72,0	28,0	
**Statut marital (N=356)**			
Seul	70,9	29,1	0,58
En union	73,6	26,4	
**Dépistage volontaire (N=356)**			
Oui	99,7	0,3	0,97
Non	72,1	27,9	
**Stade CDC (N=344)**			
A ou B	82,9	17,1	0,11
C	66,7	33,3	
**Type de protocole (N=356)**			
Efavirenz +	73,9	26,1	0,14
Efavirenz −	65,4	34,6	
**Statut vis-à-vis de la référence (N=356)**			
Référés	17,8	82,2	<10^−5^
Non référés	94,5	5,5	
**Recours au traitement traditionnel depuis l'initiation aux ARV (N=348)**			
Oui	24,0	76,0	0,02
Non	91,9	8,1	
**Variables quantitatives**	**Observants**	**Non-observants**	**P-value**
**Age (années, N=356)**			
Premier quartile	33	35	0,01
Médiane	39	43	
Troisième quartile	46	51	
**CD4 à l'initiation (Cellules/ml, N=342)**			
Premier quartile	41	41	0,58
Médiane	105	96	
Troisième quartile	166	142	
**Durée du traitement (en mois, N=356)**			
Premier quartile	14	15	<10^−3^
Médiane	21	30	
Troisième quartile	31	45

**Tableau 3:** Observance déclarée selon les caractéristiques socio-démographiques et clinico-biologiques-Unité de Prise en Charge du VIH/SIDA de Dschang, Ouest Cameroun

Variables qualitatives	Observants (%)	Non-Observants (%)	P-value
**Sexe (N=356)**			
Masculin	91,7	8,3	0,26
Féminin	87,6	12,4	
**Statut marital (N=356)**			
Seul	89,8	10,2	0,45
En union	87,4	12,6	
**Dépistage volontaire (N=356)**			
Oui	99,9	0,1	0,85
Non	88,6	11,4	
**Stade CDC (N=344)**			
A ou B	88,6	11,4	0,60
C	88,2	11,8	
**Type de protocole (N=356)**			
Efavirenz +	89,3	10,7	0,37
Efavirenz −	92,7	7,3	
**Statut vis-à-vis de la référence (N=356)**			
Référés	86,5	13,5	0,39
Non référés	89,6	10,4	
**Recours au traitement traditionnel depuis le début du TAR(N=348)**			
Oui	24,1	75,9	0,03
Non	89,7	10,3	
**Variables quantitatives**	**Observants**	**Non-observants**	**P-value**
**Age (années, N=356)**			
Premier quartile	34	31	0,02
Médiane	40	36	
Troisième quartile	48	42	
**CD4 à l'initiation (Cellules/ml, N=342)**			
Premier quartile	42	32	0,01
Médiane	105	63	
Troisième quartile	167	118	
**Durée du traitement (en mois, N=356)**			
Premier quartile	15	8	0,00
Médiane	22	15	
Troisième quartile	35	30

**Tableau 4:** Analyse multivariée-Facteurs indépendants associés à l'observance du traitement ARV chez les patients adultes suivis depuis plus de trois mois à l'Unité de Prise en Charge du VIH/SIDA de Dschang, Ouest Cameroun

Observance calculée	OR ajusté	IC 95%	P Value
Age	0,9742	0,9386–1,0111	0,1681
Durée du traitement	1,0026	0,9762–1,0296	0,8511
Recours au traitement traditionnel depuis le début du TAR	1,0606	0,2427–4,6348	0,9376
Statut vis-à-vis de la référence	0,0105	0,0046–0,0237	0,0000
**Observance déclarée**			
Age	1,0308	0,9858–1,0778	0,1829
Durée du traitement	1,0557	1,0183–1,0945	0,0032
CD4 à l'initiation	1,0079	1,0018–1,0141	0,0117
Recours au traitement traditionnel depuis le début du TAR	0,5234	0,1753–1,5628	0,2460
- **Respect des horaires de prise du TAR:** Le niveau d'adhésion aux horaires de traitement est moyen: 20% des sujets interrogés estimaient très souvent respecter les horaires de prise du TAR; 36,9% les respectaient fréquemment; 35,1% parfois et 8% rarement ou jamais.
- **Observance thérapeutique:** Le niveau global de l′observance déclarée était relativement élevé: 80,2%. L'observance calculée était de 48,7% chez les patients mis sous TAR à l′UPEC de Dschang et de 56,9% chez les patients référés à l′UPEC de Dschang, soit un taux d′observance calculé global de 51,5%. La différence entre les 2 mesures d'observance était statistiquement significative (p<10^−5^) et le pourcentage d'accord, calculé par le rapport entre le nombre de sujets observants pour les 2 méthodes sur le nombre total d'observants déclarés était de 64,3%.
- **Raisons de non-observance:** Les raisons principalement évoquées dans les cas de non-observance étaient par ordre d'importance: oubli (22,4%), travail (19,6%), endormissement (10,4%), voyage/déplacement (8,6%), absence de nourriture (7,6%).
- **Déterminants de l'observance:** En analyse univariée : pour ce qui est de l'observance calculée (Tableau 2), les personnes observantes étaient significativement plus jeunes et plus souvent mis sous TAR par le comité thérapeutique de l'UPEC de Dschang. Par ailleurs, le niveau de non-observance augmentait significativement avec la durée du traitement. Pour ce qui est de l'observance déclarée (Tableau 3), l’âge, le taux de CD4 à l'initiation thérapeutique, la durée du traitement étaient significativement plus élevés chez les sujets observants.

Analyse multivariée : Après ajustement multiple par régression logistique sur des facteurs significativement associés à l'observance dans l'analyse univariée, seul le statut vis-à-vis de la référence était significativement lié à l'observance calculée (p<10^−4^). Pour ce qui est de l'observance déclarée, la durée du traitement (p=0,00) et le taux de CD4 (p=0,01) en début de traitement étaient significativement associés à l'observance (Tableau 4).

## Discussion

Dans notre étude la moyenne d’âge est égale à 41,28 ans avec des extrêmes de 21–74 ans. C'est la tranche d’âge des sujets en activité qui est la plus affectée (66,6%) Ce résultat est conforme aux données de l'ONUSIDA [20].

Nous avons observé une prédominance féminine (71,1%) dans la cohorte. Ce résultat est similaire à ceux de Nachega et al en Afrique du Sud [21] et d'Oumar AA et al au Burkina Fasso [22].

Une fraction non négligeable (14,2%) résidait à l'extérieur du District de Santé. Or dans cette région, chaque DS dispose d'un service de prise en charge des cas. La crainte de la stigmatisation, la protection de la confidentialité de la séropositivité dans la communauté et même le degré de confidentialité du centre de soins, pourraient justifier le recours aux soins dans un autre DS.

Le niveau d'observance déclarée était significativement plus élevé que le niveau global de l'observance calculée (80,2% vs 51,5%, p<10^−5^). Bien que l'observance calculée soit a priori plus objective que l'observance déclarée, les résultats de son estimation devraient être considérés avec prudence pour 2 raisons: premièrement, la paramètres de qualité de la tenue des registres de la pharmacie ne sont pas connus et peuvent avoir une incidence sur le numérateur du ratio de l'observance calculée; ensuite, il est possible pour un malade ayant effectué un déplacement, de s'approvisionner en ARV auprès d'un centre de traitement situé dans une autre région en cas de rupture en ARV (approvisionnement de sauvetage) et dans ce cas, cet approvisionnement ne sera pas rapporté dans le registre de sortie de la pharmacie de son UPEC d'origine.

Les déclarations des patients se sont avérées propices à l′évaluation de l′observance dans une cohorte Sénégalaise [17]. Au final, seuls les dosages biologiques nous auraient permis d'apprécier le niveau réel d'observance au TAR dans cette cohorte de patients.

Les patients référés étaient moins observants que les non-reférés. Nous avons retrouvé un lien statistiquement significatif entre la durée du traitement, le taux de CD4 à l'initiation thérapeutique et l′observance. Abellan et coll [23] observaient que les individus avec un taux de CD4 élevé en début de traitement, avaient une meilleure observance par rapport à ceux ayant un taux bas (p=0,02). Par contre, à l'opposé des tendances rapportées par d'autres auteurs en contexte africain [17, 24–26], dans cette cohorte, l'observance s'améliore au fil du temps. Ceci traduit bien l'effet du counselling continu dans l'accompagnement psycho-social des patients sous TAR. L’âge et le sexe n'ont pas été associés à l'observance, comme rapporté par certains auteurs [27, 28]. Contrairement à Tanon et coll, nous n'avons pas pu établir une association significative entre le stade clinique et l′observance [29]. De même, l'influence des tradipraticiens n'a pas été associée à la non-observance comme l'atteste des études sénégalaise et européenne [24, 27–29].

Les barrières à l'observance étaient surtout l'oubli, le travail, l'endormissement, la mobilité (voyage), l'absence de nourriture. La rupture de stock à la pharmacie est la 7ème cause de non-observance et la prescription inadéquate occupe le 9ème rang parmi les raisons d′inobservance. Dans une enquête longitudinale en milieu hospitalier à Bamako, Oumar et al rapportaient que l'oubli constituait le principal facteur de mauvaise observance [22] au TAR. De tels résultats diffèrent de ceux observés dans les pays du Nord o[ugrave] les motifs liés aux médicaments, ont été rapportés comme étant la première cause de non-observance [27, 28, 30]. L'engagement du patient lui-même à suivre son traitement semble être le principal déterminant de l'observance, suivi des facteurs institutionnels comme l'illustre l'effet des ruptures de stocks en médicaments et des prescriptions inappropriées sur l'observance. Le rôle prédictif de la composante socio-économique sur la non-observance est minoré dans cette cohorte, sans doute á cause de la politique nationale de la gratuité des ARV visant à assurer l’équité d'accès pour tous les nécessiteux au TAR.

### Limites de l’étude

Une première limite de ce travail relève a priori de sa problématique: l’étude de l'observance. Il n′existe pas de méthode de référence en matière d′observance [31]. La littérature recommande de recourir à au moins deux méthodes, dont l′une doit reposer sur les déclarations du patient [32]. Les méthodes biologiques telles que la mesure de la charge virale ou le dosage des anti-protéases auraient garanti la validité de notre questionnaire [33, 34]. La question de la fiabilité des réponses du malade dans ses dires se pose donc avec une certaine acuité.

Afin de limiter les biais de mémorisation, nous avons évalué auprès de nos patients, l'observance au cours des sept et des dix jours précédents l'enquête, du mois dernier, et depuis la mise en route du traitement antirétroviral. Ce qui n'exclut cependant pas la possibilité de biais de mémorisation (perte d′information de la part du patient concernant le respect de sa prescription depuis la mise sous TAR. Par ailleurs, la collecte de données ne se faisant pas en insu, les patients en présence de l'enquêteur sont plus sujets à faire de fausses déclarations. Enfin, les limites inhérentes au type d’étude (transversale) et à la mesure utilisée pour le calcul des taux d′observance (moyenne) masquent le dynamisme de l′observance dans le temps

## Conclusion

La discordance observée entre les résultats des deux méthodes utilisées pour estimer l'observance, tout en soulevant les contraintes liées à l’évaluation de l'observance thérapeutique, souligne l'importance des méthodes biologiques. Le comptage des médicaments restants est une méthode simple et peu couteuse qui pourrait apporter des précisions sur le niveau d'observance au TAR, indépendamment des aléas liés au système d'approvisionnement des ARV dans ce contexte. Les principaux déterminants de l′observance dans cette UPEC sont le taux de CD4 à l'initiation thérapeutique, la durée du suivi, le statut vis-à-vis de la référence et les facteurs individuels constituent les principales barrières à l'observance.

Dans cette UPEC, un accent devrait être mis sur les consultations d’éducation thérapeutique et le suivi psycho-social des patients sous TAR. En particulier, la sensibilisation régulière à l'observance thérapeutique au cours des visites médicales de routine et l'utilisation des SMS de rappel pour les moins observants pourraient contribuer à améliorer l'observance des patients.

Compte tenu de l'importance du suivi médical, les mesures d'accompagnement à l'observance (entretiens de motivation et la thérapie comportementale) doivent être poursuivies, la gestion de stocks des médicaments améliorée et les capacités des professionnels de la santé renforcées.

## Matériel additionnel

Matériel additionnel: Questionnaire d’évaluation du niveau de connaissances relatives à la transmission et la prévention du VIH
